# Cases Illustrating and Confirming the Remedial Power of Inhalation of Iodine and Conium in Tubercular Phthisis, and Various Disordered States of the Lungs and Air-Passages

**Published:** 1834-04-01

**Authors:** 


*Cases illustrating and confirming the Remedial Power of Inhalation of Iodine and Conium in Tubercular Phthisis, and various Disordered States of the Lungs and Air-Passages.* By Sir Charles Scudamore, M.D., F.R.S., &c. *Second Edition.*—London, 1834. 8vo. pp. 227.

Of the occasional usefulness of inhalation in diseases of the lungs, we, in common with all who have witnessed such experiments, have no doubt; but the general testimony in favour of the practice is by no means so enthusiastic as that borne by Sir Charles Scudamore. Unprejudiced and competent judges still testify its utter inadequacy to do more than allay irritation in some cases; while in others it has proved a source of excitement that has been found highly injurious, and by no means controllable: in the majority of instances, however, its inertness in respect of permanent effects has been demonstrated to a degree deterring from repetitions deemed useless. "The rationality of applying some remedial agent in a direct manner to the seat of diseased action, in certain conditions of pulmonary and bronchial disease, has been admitted and acted upon for many past ages." Thus says the author: to whom it may be replied, that the difficulty of producing any enduring and beneficial effects has been admitted and acted upon for a good part of that time. In some bronchial affections, the advantages obtainable by such agents may reasonably be hoped for to a greater extent than in tubercular excavations of the lungs; for in those cases the disorganization, if any, is rarely to an extent incompatible with the patient's existence. While, in the cavernous ulceration of tubercular phthisis, the mischief is seldom confined exclusively to the cavity. Do we not continually see tubercles in every stage, from the miliary one to maturation, complicated with other diseases of the respiratory organs? There is no greater mistake, and no one more frequently committed, than the losing sight of the fact that phthisis is not merely a local disease. The tendency of this error may have retarded our advancement in the *methodus medendi* of this reproach to our art, which suffers as much from its supposed infallibility by one sect, as it does from the ignorant scepticism of another.

To many readers, Dr. Scudamore's formulæ for inhalation will be acceptable.

"As, by mixing the tincture of iodine with water, the iodine itself separates into flakes which become precipitated, and as 7000 parts of water are required for its solution, I found it expedient to form a preparation which should be uniform, and preserve its transparency when united with water in any proportions. This admixture is effected by adding together iodine, hydriodate of potash, distilled water, and alcohol. The following is the formula which I prefer on commencing with the tincture of iodine inhalation:

℞. Iodin. gr. v.

Potassæ Hydriodat. gr. iij.

Aquæ distillat. ℥v.

Alcoholis, ʒij.

Tinct. Conii,* ʒvi. M. fiat mistura.

"I found it expedient to use the smallest proportion of the hydriodate which would serve the purpose of rendering the iodine soluble, and not enough to engage much of the iodine itself. The addition of the tincture of conium is important; as, together with its distinct operation as a sedative, it softens the action of the iodine; and this property of diminishing the sharpness of the iodine, during the process of inhaling, is more effectually produced by the previous combination of all the ingredients; but the mixture should not be long kept, as in that case the iodine would undergo considerable change, and its power become too much reduced.

* "A saturated tincture."

Also, when it is desired to have the iodine solution in the most active state, the conium, if mixed with it at all, should be added only at the time of using the inhalation.

"The following are the other medicinal substances which I have used for the purposes of inhalation:

"A saturated solution of pure chlorine gas in distilled water.

"A saturated tincture of stramonium, prepared from the dried leaves and stalks.

"A saturated tincture of belladonna, prepared from the dried leaves.

"A saturated tincture of the lobelia inflata.

"A spirituous tincture of ipecacuanha, prepared from the roots.

"A saturated tincture of digitalis.

"Hydrocyanic acid, of the specific gravity ·992.

"The pure sulphuric æther." (P. 8.)

The following observations are judicious:

"For the relief of severe pain I should give tincture of opium, or the powder or extract, to a patient with whom opium did not disagree, in preference to any other preparation; having most reliance on all the properties of opium combined, where I wish to prescribe this medicine as an anodyne. We well know also in how remarkable a degree great pain modifies the effects of opium; so that an individual who would be disordered in the most inconvenient manner by opium, if taking it for the purpose of procuring sleep, could have recourse to it in free doses, with every good result, when using it as a remedy for severe pain." (P. 52.)

Ignorance of these points well explains the odium which opium often unjustly incurs for "disagreeing." Numerous opportunities and ample observation have convinced us that opium never produces any but the best effects, when administered in efficient doses for the relief of severe pain, or as an auxiliary to bloodletting in inflammation.

We copy one of the cases added in the present edition, as a fair sample of our author's style and method of treatment:

"CASE III. *Very irritable cough; much soreness of the larynx on pressure, and which, in conjunction with the nature of the expectoration, rendered it probable that there was ulceration of the mucous membrane.*

"A lady, aged fifty-six, of slight figure and delicate constitution, had been out of health for two years, occasionally affected with an intermittent, and always complaining of much debility. She caught a severe cold; this was speedily followed by cough, which fixed itself in a very troublesome manner. I found her complaining of much soreness and tenderness to pressure on the larynx, just below the thyroid cartilage. She stated that her troublesome cough had continued, without relief from any treatment, for six months. Eight blisters at least had been applied over the affected part, and various medicines administered. Every afternoon, at about the same hour, a paroxysm came on of incessant cough, attended with the most remarkable difficulty of breathing, suspending the power of all bodily exertion; and this state of suffering continuing till evening, she was rendered absolutely prostrate with languor. She had a quick, weak pulse. Her nights were restless, and, towards the morning, perspiration was considerable. There was much expectoration, of decidedly purulent appearance, frequently mixed with blood; and the tests which I employed convinced me of the fact of purulent secretion. I thought that there was strong evidence of an ulcerative process having been established in the mucous membrane of the trachea.

"I directed, at first, an inhaling mixture composed of the saturated tincture of conium, tincture of ipecacuanha, and hydrocyanic acid; of the first medicine thirty minims, of the second twenty, and of the last two, for each inhalation, adding two of the hydrocyanic acid, for the latter part of the process; this agreed perfectly and was useful. After a few days, having more confidence in the curative agency of the iodine than any other remedy, and as the tracheal irritation was diminished, I did not delay longer the trial of the compound iodine mixture as at p. 9. Of this, ℥iss. was, as usual, divided into two doses, and this was used three times a day. It was gradually increased to five drachms for each inhalation. She considered that the blisters weakened her without rendering her any countervailing benefit. I applied the cantharides solution (p. 90,) with an excellent effect, and it was repeated occasionally. I prescribed the sarsaparilla mixture with alkali to be taken with milk, and this was afterwards changed for the sulphate of quinine draught. Acetate of morphia was given at night, in small doses, with great advantage. The diet was supporting, and the dinner beverage consisted of port wine and water, or sherry and water, as she might choose. The case prospered from the first moment. The inhalation invariably agreed, and the occasional aggravation of cough during the process was fully compensated by the increased freedom of expectoration, and the subsequent comfortable relief of the cough and the breathing. The appearance of the expectoration gradually improved, and at the end of two months the cure was complete.

"*Observations.* It is probable that practitioners not acquainted with the useful properties of the iodine and conium inhalation, would have apprehended injurious irritation from the direct application of this stimulant; but I entered on the treatment with confidence, and happily was not disappointed by the result. This lady had so much despaired of relief, from the failure of all previous means, that she had almost resolved to avoid any new measures, when I was consulted. The direct action of the iodine changed the function of the mucous membrane of the trachea, and induced healthy action. If not in a state of actual ulceration, it was bordering on this condition. The effect of the liquid preparation of cantharides deserves comment. The counter-irritation was decidedly beneficial, and the patient did not object to the application, as she had done to the regular blister-plaster. I do not propose the use of this liquid as calculated to supersede that of the plaster, which, in most instances, excites a higher degree of action, and therefore will often prove the more valuable remedy; but there are many occasions on which it proves very convenient and preferable, on account of the facility of its application, the great promptness of its action; its odour being rather agreeable than otherwise; and the unpleasant qualities of a plaster being avoided." (P. 164.)

Should any one do us the honour of appealing from the judgment of Sir C. Scudamore to ours, we should be willing to confess that in phthisis inhalation may be commendably employed, though the chance of cure be small; but that in bronchitis, and some other diseases, it is an excellent remedy: in short, to use the expression of Dr. Mudge, it is "a radical cure for a catarrhous cough."

